# Association between the -159C/T polymorphism in the promoter region of the CD14 gene and sepsis: a meta-analysis

**DOI:** 10.1186/s12871-017-0303-9

**Published:** 2017-01-25

**Authors:** Qin Wu, Xiaomeng Xu, Jianan Ren, Song Liu, Xuelian Liao, Xiuwen Wu, Dong Hu, Gefei Wang, Guosheng Gu, Yan Kang, Jieshou Li

**Affiliations:** 1 Department of General Surgery, Jinling Hospital, Medical School of Nanjing University, 305 East Zhong Shan Road, Nanjing, 210002 China; 2 Department of Critical Care Medicine, West China Hospital, Sichuan University, Chengdu, Sichuan China

**Keywords:** CD14-159C/T, Polymorphism, rs2569190, Sepsis, Susceptibility, Mortality

## Abstract

**Background:**

The association between CD14-159C/T polymorphism and sepsis has been assessed but results of current studies appeared conflicting and inconstant. This analysis was aimed to determine whether the CD14-159C/T polymorphism confers susceptibility to sepsis or is associated with increased risk of death from sepsis.

**Method:**

The authors conducted a comprehensive search of PubMed, EMBASE, ISI Web of Science, Cochrane library, ScienceDirect, Wiley Online Library and CNKI databases according to a prespecified protocol. Language limits were restricted to English and Chinese. Two reviewers independently selected the articles and extracted relevant data onto standardized forms. Disagreements were settled by discussion and suggestions from senior consultants. The strength of association were evaluated by odds ratio (OR) and 95% confidence interval (CI). Studies failed to fit the Hardy-Weinberg-Equilibrium were excluded.

**Results:**

The research identified a total of 2317 full-text articles of which 14 articles met the predefined inclusion criteria. Meta-analysis was performed for allele frequency of C versus T, as well as genotypes CC + CT versus TT (dominant model), CC versus TT + CT (recessive model), CT versus TT and CC versus TT (additive model). All control samples were in Hardy-Weinberg proportion. No significant association between CD14-159C/T polymorphism and sepsis susceptibility or mortality were detected in the overall population. Nonetheless, subgroup analysis of Asian ethnicity revealed significant association between the CD14-159C/T polymorphism and susceptibility to sepsis in additive model (CC versus TT: OR = 0.52, 95% CI 0.29–0.92, *p* = 0.03) and recessive model (CC versus CT + TT: OR = 0.50, 95% CI 0.30–0.84, *p* = 0.009). Of note, three out of the five papers included in the subgroup focused exclusively on burn ICU patients.

**Conclusions:**

This meta-analysis demonstrated that CD14-159C/T polymorphism is likely to be associated with susceptibility to sepsis in Asian population, especially for the TT genotype. However, bias may rise for etiologic reasons because the majority of subjects in the subgroup came from burn ICU. CD14-159C/T polymorphism is not relevant to sepsis mortality in any genetic models, regardless of the ethnicities. Due to the exploratory nature of the study, no adjustment for multiple testing was adopted, and therefore the results should be interpreted with precaution. Well-designed studies with larger sample size and more ethnic groups are required to further validate the results.

**Electronic supplementary material:**

The online version of this article (doi:10.1186/s12871-017-0303-9) contains supplementary material, which is available to authorized users.

## Background

Sepsis, a complex clinical syndrome due to a systemic inflammatory response to bacteria and/or bacterial products, is imposing a huge burden on modern health care systems [1, 2]. Individual response to sepsis is determined by many factors, including the virulence of the organism and the patient’s coexisting conditions [3].

With the sequencing of human genome and the recognition of the degrees of genetic variation, it has become clear that an individual’s genetic makeup is likely to have impact on the incidence as well as outcome of sepsis [4, 5]. As is witnessed by the recent decades, an explosion of research occurred to address the effect of genetic predisposition on the development and course of sepsis, and a single nucleotide polymorphism (SNP) in CD14 with a frequency higher than 1% in the population succeeded to draw global attention [6].

CD14 plays a crucial role in sepsis. CD14 is involved in LPS recognition and mediates the activation of monocytes/macrophages in gram-negative bacterial infection. In addition, it is also claimed that LPS is associated even with the deterioration of gram-positive bacterial infection because products from such bacteria induce hypersensitivity to LPS [7]. Additionally, animal study demonstrated that expression of CD14 interacted with mortality of mice treated with endotoxin [8]. CD14-159C/T (rs2569190) is a functional polymorphism located in the 5′UTR of the promoter region of *CD14* gene, counting from the transcription start site, with a potential role of decreasing or increasing gene expression in process of sepsis [9]. Thus, it is necessary to identify whether the genetic heterogeneity in CD14 influences the response to bacterial infection.

Several studies tried to establish an association between CD14 polymorphism and sepsis [10, 11]. However, varied conclusions have been obtained in different studies. Our purpose in the current work is to determine whether this variant in CD14 is associated with an increased risk of sepsis or higher sepsis-related mortality by means of a human genome epidemiology review including a meta-analysis of previous data.

## Methods

### Literature search strategy

This meta-analysis was performed according to the recommendations of the Meta-analysis of Observational Studies in Epidemiology (MOOSE) guidelines [12]. Two independent investigators performed a systematic review of English-language and Chinese-language literature in PubMed, EMBASE, ISI Web of Science, Cochrane library, ScienceDirect, Wiley Online Library and CNKI databases with the following terms: (CD14 OR CD14-159 OR CD14-159C/T OR rs2569190) AND (sepsis OR septic shock OR severe sepsis OR severe burn OR major trauma). In addition, we also searched the references of original research reports and review articles (last search: 2016.09.22). By using the MEDLINE option ‘related articles’, we tried to find all studies potentially relevant. Both investigators have received training in literature search, statistic and evidence-based medicine in Nanjing University. References of the selected publications were manually scanned to identify other relevant studies.

### Inclusion and exclusion criteria

Studies were included in our meta-analysis if 1) they were focused on the relationship between CD14-159C/T polymorphism and the susceptibility to sepsis or sepsis-related mortality, 2) they studied humans, 3) enough data of selected SNP of both patients and control groups were clearly stated to calculate odds ratio (OR) and 95% confidence interval (CI), 4) the genotype frequency of control group was in accordance with Hardy-Weinberg equilibrium (HWE) [13]. Sepsis-related mortality was defined as death caused by sepsis, severe sepsis or septic shock. For each study, HWE was evaluated using the goodness-of-fit chi-square test by our investigators. A *p* < 0.05 was considered representative of a departure from HWE.

Exclusion criteria were 1) editorials, letters, comments, practice guidelines, consensus development conferences, and book chapters 2) causes of the systemic inflammatory response syndrome (SIRS) were not reliably defined;3) duplications. Disagreements on including/excluding studies were resolved by consensus. For studies without full text or sufficient data, we contacted the corresponding authors for additional information by e-mail. If no reply or the author refused to provide the data required in this meta-analysis, the study was excluded.

### Data extraction

Using a standardized form, two investigators independently extracted data from the included studies. For each eligible study, the following characteristics were collected: authors, publication date, ethnicity, numbers of cases, sample size, allele and genotype frequencies. The frequencies of alleles were calculated for cases and controls from the corresponding genotype distributions. Both of the investigators checked the extracting data results and disagreements were settled by discussion. Senior investigators were invited to discuss if disagreement still existed.

### Statistical analysis

We recorded data from all publications with a computerized spreadsheet (Microsoft Excel). ORs and 95% CIs were calculated to estimate the association between CD14-159C/T polymorphism and susceptibility or mortality of sepsis. Pooled ORs were calculated for allele frequency comparison (C versus T), additive model (CT versus TT, CC versus TT), dominant model (CC + CT versus TT), and recessive model (CC versus TT + CT), respectively. Since the studies included are not as high-quality as RCTs, random effects models were used in each analysis whether or not heterogeneity exists. Therefore the result will be more conservative and can be interpreted in a homogenous way. The significance of pooled ORs was determined by Z-test and *p* < 0.05 was considered as statistically significant. Due to the exploratory nature of the study, *p*-values were not adjusted for multiple testing.

Statistical heterogeneity among the studies was checked by chi-square-based Q-test. An I^2^ value less than 50% indicated no significant heterogeneity. Meta-regression was adopted to determine the potential source of heterogeneity.

Sensitivity analysis was carried out by omitting one single study each time to examine the influence of individual data sets on the pooled ORs. Publication bias of literature was assessed using funnel plots and Egger’s test. An asymmetric plot suggested a possible publication bias and *P* value of Egger’s test less than 0.05 was considered statistically significant. All analyses were performed using the software program RevMan Analyses software (RevMan 5.0.17) from the Cochrane collaboration. All statistical analyses were performed with Stata 12.0 software (StataCorp, College Station, TX, USA). The statistical methodology was approved by principal investigator (Ren, J) and the statistical analysis were performed by trained investigators (Wu, Q and Xu, X).

## Results

### Characteristics of the studies

**Fig. 1:**
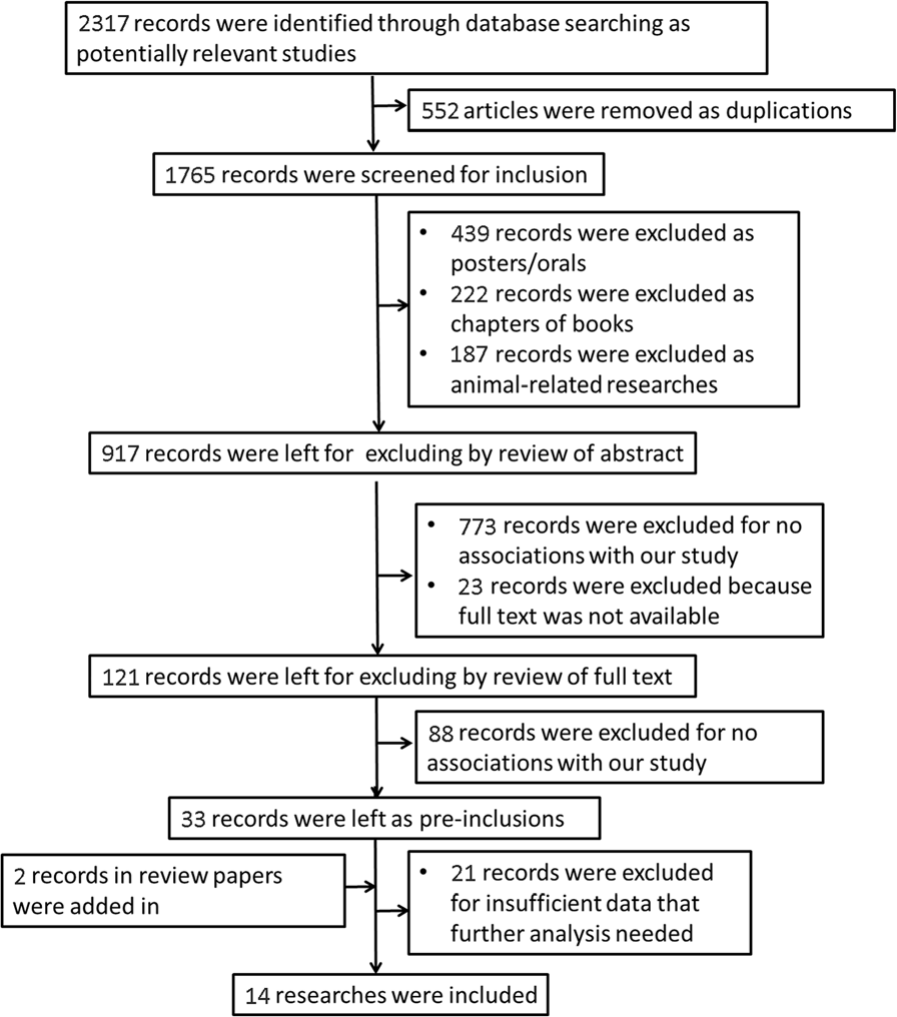
Flow chart of study identification and exclusion for the meta-analysis

The literature search identified a total of 2317 potentially relevant studies in PubMed, EMBASE, ISI Web of Science, Cochrane Central Register of Controlled Trials, ScienceDirect, Wiley Online Library and CNKI databases. As was shown in Fig. 1, 552 duplicate records were removed. Four hundred thirty-nine papers were also excluded as posters or orals, as well as 222 records obtained from books. Additionally, 109 studies conducted in animal models were dropped. Nine hundred seventeen records were analyzed by reviewing the abstracts. After reading abstracts, irrelevant studies were excluded whereas two additional papers were included manually after reading reviews. Therefore, a total of 33 papers met the primary inclusion criteria, among which 21 papers failed to provide sufficient data for calculation of OR and 95% CI and were excluded, as well as one paper [14] which deviated from HWE in controls. A total of 14 studies for the association between CD14 polymorphism and sepsis were included in the final meta-analysis.

Characteristics of included studies were summarized in Table 1. Ten studies that evaluated the effect of the CD14 polymorphisms on incidence of sepsis were ultimately analyzed in our meta-analysis, as well as nine studies that demonstrated the effect of the CD14 polymorphisms on sepsis-related mortality.

**Table 1:** Characteristics of studies included in the meta-analysis

Study	Ethnicity	SNP genotyping assay	Patient group	No. of Cases	HWE^a^	Sepsis definition	Susceptibility reported	Outcome reported
Barber, 2007 [28]	Caucasian	PCR	Burn ICU	233	Yes	No	No	Yes
Bronkhorst, 2013 [33]	Caucasian	High-resolution Melting Analysis	Trauma center	219	Yes	Yes	Yes	No
D’Avila, 2006 [30]	Caucasian	PCR-RFLP	General ICU	85	Yes	Yes	Yes	Yes
Dong, 2009 [34]	Asian	PCR-RFLP	Burn ICU	77	Yes	Yes	Yes	No
Dong, 2010 [35]	Asian	PCR-RFLP	Burn ICU	35	Yes	Yes	Yes	Yes
Fallavena, 2009 [16]	Caucasian	Sequence Analysis	ICU	514	Yes	Yes	Yes	Yes
Gibot, 2002 [22]	Caucasian	PCR-RFLP	ICU	212	Yes	Yes	Yes	Yes
Gu, 2008 [29]	Asian	PCR-RFLP	Trauma center	105	Yes	Yes	Yes	No
Heesen, 2002 [25]	Caucasian	Fluorescence-labeled Hybridization Probes	Trauma center	58	Yes	Yes	Yes	No
Hubacek, 2000 [26]	Caucasian	PCR	ICU	204	Yes	No	No	Yes
Jessen, 2007 [39]	Caucasian	PCR-RFLP	ICU	452	Yes	Yes	No	Yes
Lin, 2004 [32]	Asian	PCR-RFLP	Burn ICU	16	Yes	Yes	Yes	Yes
Nakada, 2005 [40]	Asian	PCR-RFLP	Surgical ICU	197	Yes	Yes	Yes	No
Shimada, 2011 [41]	Asian	Fluorescence-labeled Hybridization probes	ICU	224	Yes	Yes	No	Yes

^a^Hardy-Weinberg equilibrium (HWE) was evaluated using the goodness-of-fit chi-square test. *P* values were presented. *P* > 0.05 was considered representative of a departure from HWE

All studies were published in English except for three in Chinese. Nine studies were performed in Caucasian populations and five in Asian. In the studies included, sepsis was commonly defined according to the American College of Chest Physicians/Society of Critical Care Medicine guidelines [15].

### Test of heterogeneity

Heterogeneity between studies in each comparison was shown in Table 4 for susceptibility and Table 5 for mortality. Heterogeneity was insignificant (I^2^ less than 50%) in the pooled analyses of susceptibility (Table 4), whereas remarked heterogeneity existed in the evaluation of mortality in all five genetic models (Table 5). In order to evaluate the influence of the individual data set to the overall heterogeneity, every single study was omitted each time in every genetic model. As a result, the study by Fallavena et al. [16] is the major contributor to the total heterogeneity (Additional file 1: Tables S1 and S2). Meta-regression was conducted to determine the potential source of heterogeneity (Additional file 1: Table S3), and ethnicity contributes to indispensable parts of variance.

Subgroup analysis was conducted and all the subjects were stratified by ethnicity. Heterogeneity among studies in each subgroup was estimated in the five genetic models. No heterogeneity was observed in each comparison in Asian population; on the contrary, evident heterogeneity remained among Caucasian subgroup (Tables 4 and 5).

### CD14 polymorphism and susceptibility to sepsis

Ten studies reported results on susceptibility to sepsis (Table 2). The association between the CD14-159C/T polymorphism and susceptibility to sepsis was analyzed in these independent studies with 788 cases and 730 controls (Table 4). One hundred ninety-seven of 405 (48.64%) patients with the CD14-159 CC genotype group developed sepsis, as compared to 198 of 383 (51.69%) of those with CD14-159 TT genotype.

**Table 2:** Characteristics of included studies in the meta-analysis analyzing the effect of the CD14 polymorphism on incidence of sepsis

Study	SNP	Sepsis Group (n)			Control Group (n)			Distribution of alleles			
		CC	CT	TT	CC	CT	TT	Sepsis Group		Control Group	
								C	T	C	T
Bronkhorst, 2013 [33]	CD14-159C/T	22	37	20	37	68	35	81	77	142	138
Dong, 2009 [34]	CD14-159C/T	3	38	15	4	11	6	44	68	19	23
Dong, 2010 [35]	CD14-159C/T	3	9	7	4	7	5	15	23	15	17
Gibot, 2002 [22]	CD14-159C/T	19	43	28	44	52	26	81	99	140	104
Gu, 2008 [29]	CD14-159C/T	5	23	14	16	35	12	33	51	67	59
Lin, 2004 [32]	CD14-159C/T	0	4	3	2	6	1	4	10	10	8
Nakada, 2005 [40]	CD14-159C/T	15	43	28	27	42	42	73	99	96	126
D’Avila, 2006 [30]	CD14-159C/T	17	19	16	10	16	7	53	51	36	30
Fallavena, 2009 [16]	CD14-159C/T	108	172	63	49	78	44	388	298	176	166
Heesen, 2002 [25]	CD14-159C/T	5	5	4	15	22	7	15	13	52	36

*SNP* single nucleotide polymorphism

**Fig. 2:**
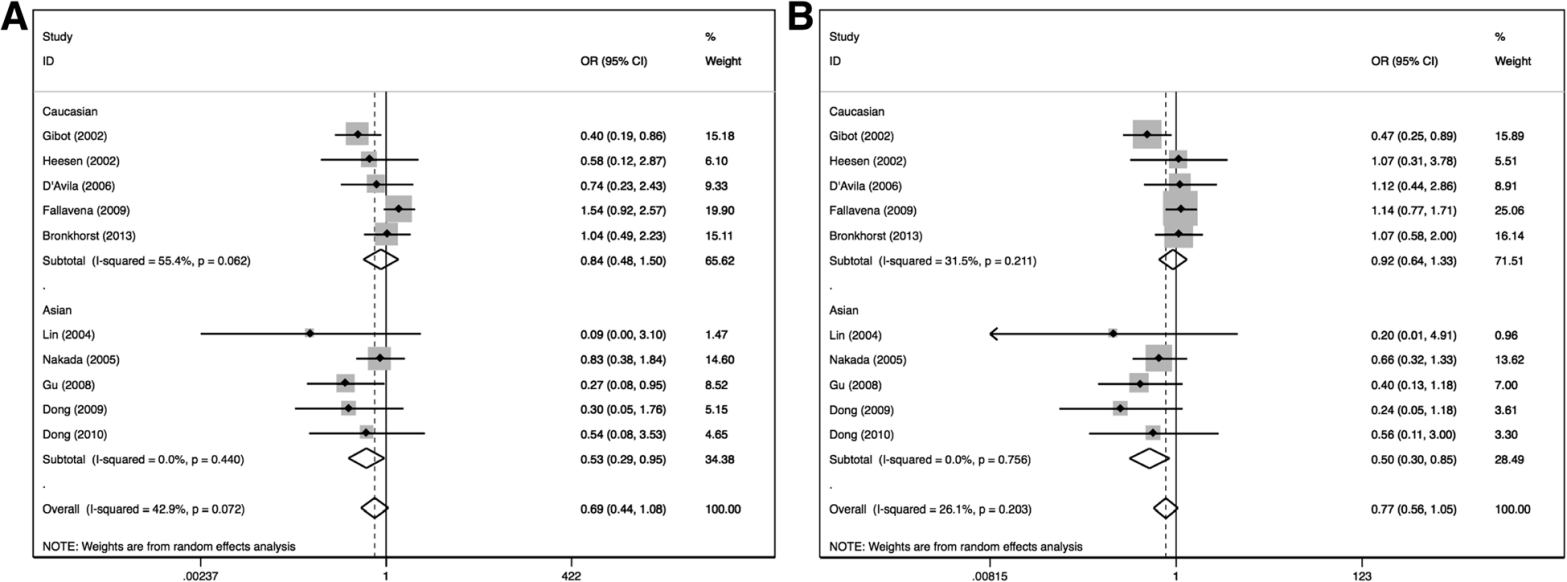
Forest plots of association between the CD14-159C/T polymorphism and susceptibility to sepsis in CC vs TT (**a**) and CC vs CT + TT (**b**) genetic models. Significant association was detected in Asian subgroups in these two models. Odds ratios (OR) with 95% confidence intervals (CI) for individual studies and the pooled OR and 95% CI were given

**Fig. 3:**
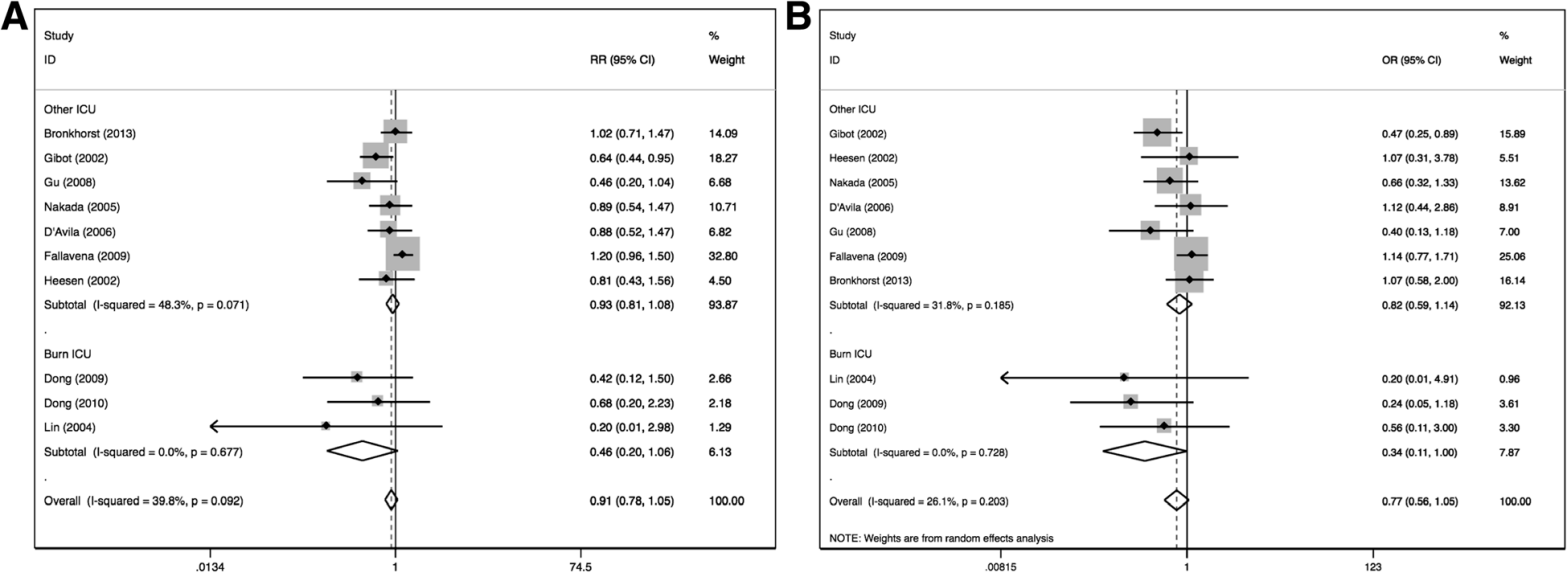
Forest plots of association between the CD14-159C/T polymorphism and susceptibility to sepsis in burn ICU sub group in CC vs TT (**a**) and CC vs CT + TT (**b**) genetic models. Significant association was detected in Asian subgroups in these two models. Odds ratios (OR) with 95% confidence intervals (CI) for individual studies and the pooled OR and 95% CI were given

No association between the CD14-159C/T polymorphism and susceptibility to sepsis was identified in any genetic modes (Table 4). However, subgroup meta-analyses indicated significant association between the CD14-159C/T polymorphism and susceptibility to sepsis in additive model and recessive model (CC versus TT: OR = 0.53, 95% CI 0.29–0.95, *p* = 0.03; CC versus CT + TT: OR = 0.50, 95% CI 0.30–0.85, *p* = 0.010). No significant association was found in other genetic models in Asian population and in Caucasian population (Fig. 2). Among patients from burn ICU, association was found in recessive model (CC versus CT + TT: OR = 0.34, 95% CI 0.11–1.00, *p* = 0.05), and board-line significant association was also observed in additive model (CC versus TT: OR = 0.33, 95% CI 0.10–1.11, *p* = 0.07) (Fig. 3).

### CD14 polymorphism and sepsis-related mortality

Nine studies contributed to our analysis of sepsis-related mortality (Table 3). One hundred forty-five of 456 (31.80%) patients with CD14-159 CC genotype died from sepsis-related diseases, compared with 257 (32.89%) patients with CD14-159 CT genotype and 98 (26.70%) patients with CD14-159 TT genotype.

**Table 3:** Characteristics of included studies in the meta-analysis analyzing the effect of the CD14 polymorphism on sepsis-related mortality

Study	SNP	Non-survivors (n)			Survivors (n)			Distribution of alleles			
		CC	CT	TT	CC	CT	TT	Non-survivors		Survivors	
								C	T	C	T
Barber, 2007 [28]	CD14-159C/T	19	18	4	51	95	46	56	26	197	187
Dong, 2010 [35]	CD14-159C/T	1	4	4	6	12	8	6	12	24	28
Gibot, 2002 [22]	CD14-159C/T	5	25	20	14	18	8	35	65	46	34
Hubacek, 2000 [26]	CD14-159C/T	21	59	17	32	52	23	101	93	116	98
Jessen, 2007 [39]	CD14-159C/T	18	33	11	74	123	55	69	55	271	233
Lin, 2004 [32]	CD14-159C/T	0	4	3	2	6	1	4	10	10	8
Shimada, 2011 [41]	CD14-159C/T	5	5	11	24	52	26	15	27	100	104
D’Avila, 2006 [30]	CD14-159C/T	17	24	9	10	11	14	58	42	31	39
Fallavena, 2009 [16]	CD14-159C/T	59	85	19	98	165	88	203	123	361	341

*SNP* single nucleotide polymorphism

**Fig. 4:**
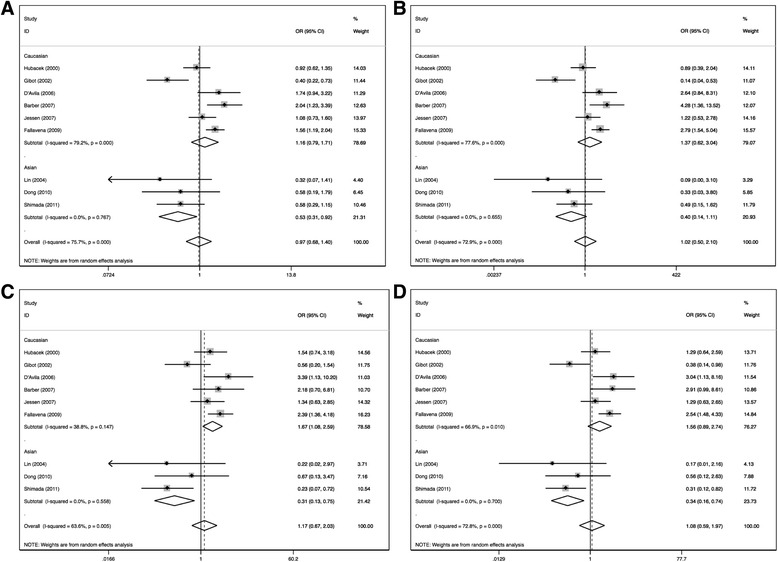
Forest plots of associations between CD14-159 polymorphism and sepsis-related mortality in allele frequency (**a**), additive model (**b** and **c**) and dominant model (**d**). Odds ratios (OR) with 95% confidence intervals (CI) for individual studies and the pooled OR and 95% CI were given

Figure 3 shows the meta-analysis of the association between the CD14-159C/T polymorphism and sepsis-related mortality in an allele frequency comparison. No association between CD14-159C/T polymorphism and sepsis-related mortality was identified in any genetic model. No further associations were observed among burn ICU subgroup. (Table 5) However, an obvious association was found between sepsis-related mortality and CD14-159C/T polymorphism in Asian subgroup in allele frequency, additive model and dominant model (C versus T: OR = 0.53, 95% CI 0.31–0.92, *p* = 0.02; CC versus TT: OR = 0.40, 95% CI 0.14–1.11, *p* = 0.08; TC versus TT : OR = 0.31, 95% CI 0.13–0.76, *p* = 0.009; CC + CT versus TT: OR = 0.34, 95% CI 0.16–0.74, *p* = 0.007), In Caucasian populations, association was identified only in TC versus TT (OR = 1.67, 95% CI .08–2.59, *p* = 0.02) (Fig. 4).

### Publication bias

Funnel plot and Egger’s test were performed to assess the publication bias of the studies and the results were shown in Tables 4 and 5. Significant publication bias was observed in the total data set in the pooled analysis of the association between CD14-159C/T polymorphism and susceptibility or mortality. After stratification by ethnicities, the bias remained remarkable in Caucasian population but disappeared in the subgroup of Asian population.

**Table 4:** Summary of pooled ORs in the meta-analysis of CD14 159C/T analyzing the effect of the CD14 polymorphism on incidence of sepsis

Genetic Model	Pooled OR	95% CI		P	Chi-square	p^#^	I^2^	P*	Statistical Power
		Lower	Upper						
CD14-159C/T									
C vs T	0.85	0.69	1.05	0.13	14.72	0.10	39%	0.048	0.155
CC vs TT	0.69	0.44	1.08	0.10	15.77	0.07	43%	0.022	0.134
TC vs TT	0.99	0.73	1.35	0.96	11.19	0.26	20%	0.024	0.102
CC + CT vs TT	0.88	0.64	1.22	0.44	13.57	0.14	34%	0.024	0.434
CC vs CT + TT	0.77	0.56	1.05	0.10	12.18	0.20	26%	0.008	0.337
CD14-159C/T in Asian population									
C vs T	0.77	0.58	1.02	0.07	3.70	0.45	0%	0.181	0.520
CC vs TT	0.53	0.29	0.95	0.03	3.76	0.44	0%	0.110	0.701
TC vs TT	1.03	0.62	1.73	0.90	4.68	0.32	14%	0.236	0.092
CC + CT vs TT	0.84	0.50	1.42	0.52	5.07	0.28	21%	0.203	0.071
CC vs CT + TT	0.50	0.30	0.85	0.01	1.89	0.76	0%	0.134	0.884
CD14-260C/T in Caucasian population									
C vs T	0.91	0.68	1.23	0.55	9.12	0.06	56%	0.387	0.050
CC vs TT	0.84	0.48	1.50	0.56	8.96	0.06	55%	0.32	0.050
TC vs TT	0.93	0.60	1.44	0.75	6.50	0.16	38%	0.035	0.067
CC + CT vs TT	0.88	0.55	1.40	0.59	8.33	0.08	52%	0.276	0.451
CC vs CT + TT	0.92	0.64	1.33	0.66	5.84	0.21	32%	0.800	0.060
CD14-159C/T in Burn ICU									
C vs T	0.69	0.40	1.17	0.17	1.17	0.56	0%	0.218	0.290
CC vs TT	0.33	0.10	1.11	0.07	0.81	0.67	0%	0.386	0.517
TC vs TT	0.98	0.41	2.35	0.97	1.61	0.45	0%	0.023	0.050
CC + CT vs TT	0.80	0.35	1.83	0.60	1.75	0.42	0%	0.023	0.082
CC vs CT + TT	0.34	0.11	1.00	0.05	0.63	0.73	0%	0.786	0.435

p^#^: *P*-value for heterogeneity test. p*: *P*-value for Egger’s test; *OR* odds ratio, *CI* confidence interval

**Table 5:** Summary of pooled ORs in the meta-analysis of CD14 159C/T analyzing the effect of the CD14 polymorphism on sepsis-related mortality

Genetic Model	Pooled OR	95% CI		P	Chi-square	p^#^	I^2^	P*	Statistical Power
		Lower	Upper						
CD14-159C/T									
Cvs T	0.97	0.68	1.40	0.88	32.94	<0.01	76%	0.164	0.314
CC vs TT	1.02	0.50	2.10	0.96	29.56	<0.01	73%	0.060	0.363
TC vs TT	1.17	0.67	2.03	0.59	21.97	<0.01	64%	0.877	0.520
CC + CT vs TT	1.08	0.59	1.97	0.79	29.44	<0.01	73%	0.695	0.525
CC vs CT + TT	0.96	0.61	1.50	0.84	20.01	0.01	60%	<0.01	0.074
CD14-159C/T in Asian population									
C vs T	0.53	0.31	0.92	0.02	0.53	0.77	0%	0.432	0.650
CC vs TT	0.40	0.14	1.11	0.08	0.85	0.65	0%	0.229	0.513
TC vs TT	0.31	0.13	0.74	0.009	1.17	0.56	0%	0.849	0.688
CC + CT vs TT	0.34	0.16	0.74	0.007	0.71	0.70	0%	0.873	0.739
CC vs CT + TT	0.75	0.29	1.95	0.56	1.22	0.54	0%	0.005	0.163
CD14-159C/T in Caucasian population									
C vs T	1.16	0.79	1.71	0.46	24.04	<0.01	79%	0.498	0.541
CC vs TT	1.37	0.62	3.04	0.44	22.36	<0.01	78%	0.392	0.631
TC vs TT	1.67	1.08	2.59	0.02	8.17	<0.01	39%	0.648	0.823
CC + CT vs TT	1.56	0.89	2.74	0.12	15.08	<0.01	67%	0.627	0.836
CC vs CT + TT	1.00	0.59	1.69	0.99	18.07	<0.01	72%	0.289	0.085
CD14-159C/T in Burn ICU									
C vs T	0.84	0.26	2.73	0.77	8.27	0.02	76%	0.027	0.268
CC vs TT	0.74	0.07	8.30	0.81	6.74	0.03	70%	0.076	0.244
TC vs TT	0.99	0.30	3.30	0.98	0.21	0.90	37%	0.134	0.065
CC + CT vs TT	0.86	0.17	4.27	0.85	5.78	0.06	65%	0.206	0.126
CC vs CT + TT	0.96	0.20	4.67	0.96	4.12	0.13	51%	0.038	0.265

p^#^: *P*-value for heterogeneity test. p*: *P*-value for Egger’s test; *OR* odds ratio; *CI* confidence interval

### Sensitivity analysis

**Fig. 5:**
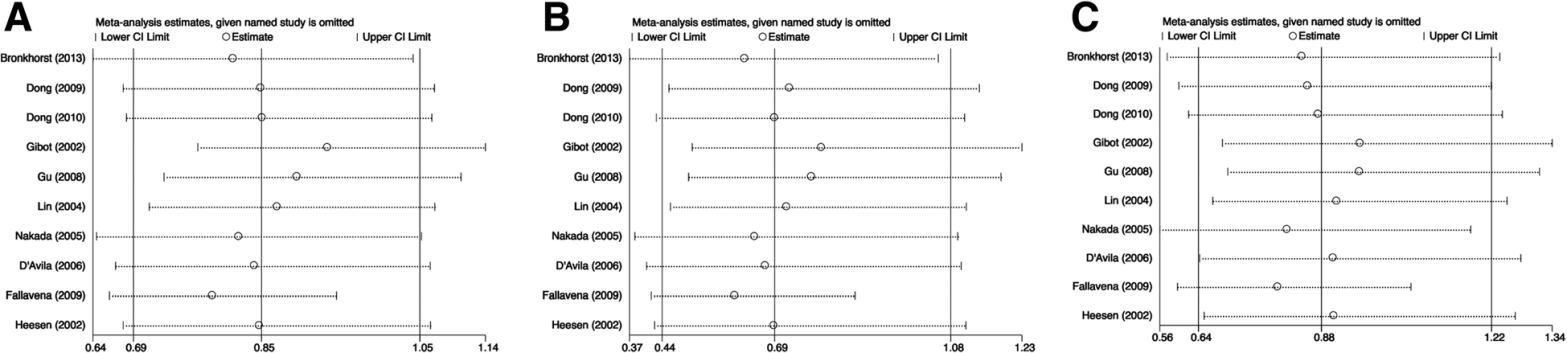
Sensitive analysis of the pooled analysis of the association of the CD14-159C/T polymorphism with susceptibility to sepsis. Odds ratios (OR) with 95% confidence intervals (CI) after omitting each individual study were given in C vs T (**a**), CC vs TT (**b**), CC + CT vs TT (**c**)

To examine the influence of the individual data set to the pooled ORs, every single study involved in this meta-analysis was omitted each time in every genetic model. For CD14-159C/T susceptibility to sepsis, after the exclusion of the study from Fallavena et al. [16], the pooled results in the following models became significant (Fig. 5): C versus T: OR = 0.79, 95% CI 0.66–0.94, *p* = 0.01; CC versus TT: OR = 0.60, 95% CI 0.42–0.86, *p* = 0.005; CC + CT versus TT: OR = 0.68, 95% CI 0.50–0.91 *p* = 0.01. Of note, this study contributed most remarkably to the heterogeneity of the pooled results focusing on susceptibility (Additional file 1: Table S1). No individual study affected the pooled OR in the analysis of mortality and in each subgroup studies.

## Discussion

Sepsis is a complex syndrome initiated by infection and is characterized by a systemic inflammatory response [17]. The preponderance of research in sepsis has focused on dissecting roles of the immune cells, innate immune regulation, cytokines, and coagulation factors in response to varying infectious and inflammatory mediators [11, 18]. This meta-analysis mainly focused on the associations between CD14-159C/T polymorphism and susceptibility to sepsis or sepsis-related mortality.

CD14 is a major component involved in the LPS receptor complex along with toll-like receptor 4, acting as a key factor in immune cell recognitions [19]. The soluble form of CD14 is essential for TLR2 or TLR4 responses in monocytes, macrophages, neutrophils, and hepatocytes [19–21]. Increased serum CD14 levels have been shown to correlate with shock and greater mortality in patients with gram-positive and gram-negative bacterial infections [22]. Polymorphisms of CD14 have been widely studied in inflammatory bowel disease and results showed a varied susceptibility in different populations, indicating that genotype frequencies vary between the populations [23, 24].

CD14-159C/T has been identified to correlate with CD14 serum levels [25–27]. A mass of studies reported the associations between CD14 SNP and incidence of sepsis, or sepsis-related mortality [10, 28]. However conclusions from different studies are controversial [29, 30]. Thus, in the present study, we identified 15 genetic association studies and used meta-analysis with the genetic model to evaluate the association of CD14-159C/T polymorphism with sepsis.

On one hand, the study investigated relations between CD14-159C/T polymorphism and susceptibility to sepsis. In the total data set, the pooled analysis exhibited no significant association between the polymorphism and susceptibility. However, in the pooled analysis focusing on susceptibility, after omitting the most significant contributor of heterogeneity [16], the results suggested that T allele and TT in additive model (CC versus TT) and dominant model (CC + CT versus TT) tend to be associated with increased susceptibility. Subgroup analysis among Asian population illustrated that the T allele and the CD14-159 TT genotype was related to susceptibility to sepsis. Results from sensitivity analysis confirmed this conclusion, and test of heterogeneity in this subgroup indicated a high constancy, indicating the steadiness of the finding. Studies verified that carriage of the T allele is associated with an increased risk for developing severe sepsis after major trauma and severe burns [31, 32]. The potential explanation for this is that the CD14-159 TT genotype reduces the promoter activity of CD14, resulting in decreased TLR2/4 downstream signaling [33]. However, interpretation of this result should be taken with precaution since the sample size of the Asian subgroup was relatively small, involving only 654 subjects, and the strength of this finding was therefore attenuated. On the other hand, the study evaluated the associations between CD14 polymorphism and sepsis-related mortality. As our study revealed, CD14 polymorphism was proved irrelevant to sepsis-related mortality in total population. Although data among Asian population reached statistical significance, the significance disappeared in subsequent sensitive analysis upon the removal of the study by Shimada et al., which counted for the largest sample size, indicating that the significance was derived from bias rather than the accumulative effects of every study in the subgroup.

The study by Zhang et al. found, that the CD14-159C/T polymorphism may not be a significant susceptibility factor in the risk of sepsis and mortality. Furthermore, only weak associations were observed in Asian populations and septic shock patients, which is similar with our conclusion [10]. Although both of us concentrated on the same topic, we still have some significant differences. First of all, different studies were included in the current analysis. In our manuscript, we included 15 papers while 17 papers were included in Zhang’s paper. In Zhang’s paper, the latest research was done in June 2012 whereas our study retrieved publications till up to September 2016 and the latest include d article was published in 2013. In addition, we excluded the paper that did not fit for the Hardy-Weinberg equilibrium while two reports deviated from HWE were finally included in the 17 papers in Zhang’s paper [10]. Secondly, in Zhang’s paper, they used three out of five genetic models to evaluate the association while in our manuscript we used all five genetic models. Thus, our study adds relevant information to the influence of this genetic variant on the outcome of sepsis. Finally, comparing the reports included in Zhang’s paper and our study, the current study included five additional papers in which four were conducted in burn ICU [28, 32, 34, 35]. This difference may lead to the discrepancy in our results. Therefore a subgroup analysis regarding to burn ICU was conducted. As a result, marginal difference was observed in additive model and recessive model. Burn ICU has several features, including disordered systematic immunological function, increased vulnerability to wound infection [36] and altered metabolic response [37]. These features may interact with the underlying mechanism in the pathogenesis of sepsis in general ICU and Burn ICU. Therefore our result may be more suitable in burn patients. However, a comprehensive understanding of the mechanism still calls for further clinical, preclinical and basic scientific studies.

Still there are several limitations in the present study. First and foremost comes the small sample size and lack of patient-level details of some studies included in the meta-analysis. Genetic association studies, as a branch of etiology, are undoubtedly an innovative strategy to explore the latent cause of certain diseases. Yet despite the rapid advance in the recent decades, when compared to classical observational epidemiologic studies, genetic association studies are still relatively rare and the sample size are generally small, which may partially attribute to a relatively higher requirement of technicians and facilities. In the case of our analysis, studies reporting blind genotyping and adjustment for confounders were few. In addition, we excluded papers whose genotype deviated from Hardy-Weinberg equilibrium and statistically assessing publication bias. Second, this study focused only on the CD14 polymorphism regardless of the likelihood that other genetic variations may also influence sepsis risk and mortality [38]. Besides, diverse study populations were included in our study, and different treatment for sepsis was used in different studies, thus contributing to differences in patients’ outcome from sepsis. Many studies had potential biases that influenced the patient prognosis. A number of papers selected involved burn and trauma patients [28, 34]. Although subgroup analysis was employed in this study to minimize the bias derived from ethnicity, and the heterogeneity in the Asian subgroup seemed statistically insignificant, interpretation of the results from the total and Caucasian populations still calls for precaution. Thirdly, the current study is an exploratory study without correction for multiple testing. The study is aimed to generate a hypothesis by means of meta-analysis rather than to test or confirm it. The aim of our meta-analysis was to generate a hypothesis rather than to test or confirm it. In this setting, multiple testing is not preferred. Therefore, our findings should be interpreted with precaution. Finally both community-acquired and nosocomial sepsis were included resulting in a heterogeneity.

## Conclusions

In conclusion, the present study revealed a potential association between CD14-159C/T polymorphism and susceptibility to sepsis in the Asian population, especially the CD14-159 TT genotype. However, due to the exploratory nature of the current study, our findings should be interpreted with precaution. Establishment of a definite association between sepsis and CD14 polymorphisms requires further trials with larger patient numbers and optimized methodology. Future trials that examine the effect of other polymorphisms are also needed to consider the effect of multiple polymorphisms.

## Additional file

Additional file 1: Supplementary Material. **Table S1.** Heterogeneity of pooled analysis focusing on the susceptibility of sepsis after omitting each study included. **Table S2.** Heterogeneity of pooled analysis focusing on the mortality after omitting each study included. **Table S3.** The percentage of heterogeneity contributed by Ethnicity and publication year. (DOCX 70 kb).

## Abbreviations

CI: Confidence interval
HWE: Hardy-Weinberg equilibrium
MOOSE: The Meta-analysis of Observational Studies in Epidemiology
OR: Odds ratio
SIRS: Systemic inflammatory response syndrome
SNP: Single nucleotide polymorphism
