# Supplementary material for: Association between the -159C/T polymorphism in the promoter region of the CD14 gene and sepsis: a meta-analysis
Source: BMC Anesthesiol. 2017 Jan 25;17:11. doi: 10.1186/s12871-017-0303-9 (PMC5264438; doi:10.1186/s12871-017-0303-9)
Supplement: Supplementary file 1 — Supplementary Material. Table S1. Heterogeneity of pooled analysis focusing on the susceptibility of sepsis after omitting each study included. Table S2. Heterogeneity of pooled analysis focusing on the mortality after omitting each study included. Table S3. The percentage of heterogeneity contributed by Ethnicity and publication year. (DOCX 70 kb). [file 12871_2017_303_MOESM1_ESM.docx]

**Supplementary Material**

Table S1. Heterogeneity of pooled analysis focusing on the susceptibility of sepsis after omitting each study included.

|  | C vs T | CC vs TT | TC vs TT | CC+CT vs TT | CC vs CT+TT |
| --- | --- | --- | --- | --- | --- |
| **Total I^2^ (%)** | 39 | 43 | 20 | 34 | 26 |
| **I^2^ (%) by omitting** |  |  |  |  |  |
| Bronkhorst, 2013 | 44 | 48 | 28 | 41 | 29 |
| Dong, 2009 | 45 | 45 | 27 | 41 | 19 |
| Dong, 2010 | 45 | 49 | 28 | 41 | 33 |
| Gibot, 2002 | 18 | 31 | 22 | 27 | 8 |
| Gu, 2008 | 32 | 36 | 14 | 27 | 23 |
| Lin, 2004 | 37 | 44 | 18 | 32 | 30 |
| Nakada, 2005 | 45 | 49 | 16 | 37 | 32 |
| D'Avila, 2006 | 46 | 49 | 16 | 37 | 32 |
| Fallavena, 2009 | 0 | 0 | 0 | 0 | 2 |
| Heesen, 2002 | 45 | 49 | 17 | 36 | 33 |

Table S2. Heterogeneity of pooled analysis focusing on the mortality after omitting each study included.

|  | C vs T | CC vs TT | TC vs TT | CC+CT vs TT | CC vs CT+TT |
| --- | --- | --- | --- | --- | --- |
| **Total I^2^ (%)** | 74 | 72 | 68 | 73 | 55 |
| **I^2^ (%) by omitting** |  |  |  |  |  |
| Barber, 2007 | 72 | 70 | 70 | 74 | 46 |
| Dong, 2010 | 76 | 74 | 71 | 76 | 59 |
| Gibot, 2002 | 67 | 64 | 69 | 72 | 30 |
| Hubacek, 2000 | 77 | 75 | 72 | 76 | 53 |
| Jessen, 2007 | 77 | 75 | 71 | 76 | 60 |
| Lin, 2004 | 75 | 73 | 70 | 75 | 58 |
| Shimada, 2011 | 75 | 73 | 61 | 70 | 60 |
| Watanabe, 2012 | 76 | 73 | 64 | 73 | 55 |
| D'Avila, 2006 | 75 | 73 | 66 | 73 | 60 |
| Fallavena, 2009 | 69 | 63 | 58 | 64 | 60 |

Table S3. The percentage of heterogeneity contributed by Ethnicity and publication year

|  | C vs T | CC vs TT | TC vs TT | CC+CT vs TT | CC vs CT+TT |
| --- | --- | --- | --- | --- | --- |
| **Susceptibility** |  |  |  |  |  |
| Ethnicity | -5.14% | 3.93% | -46.51% | -27.09% | 35.86% |
| Year | 36.08% | 30.84% | -34.80% | 4.45% | 73.03% |
|  |  |  |  |  |  |
| **Mortality** |  |  |  |  |  |
| Ethnicity | 25.14% | 13.05% | 51.23% | 43.71% | -17.01% |
| Year | -16.12% | -17.68% | -19.13% | -23.17% | 14.79% |
